# The Removal from the Esophagus of a Plate of False Teeth Embedded for Eighteen Years, by Means of the Esophagoscope

**Published:** 1913-10

**Authors:** D. Braden Kyle

**Affiliations:** Philadelphia


### The Removal from the Esophagus of a Plate of False Teeth Embedded for Eighteen Years, by Means of the Esophagoscope.

Dr. D. Braden Kyle, Philadelphia: This foreign body which I present was very difficult to locate, in spite of its size and shape, on account of the granulation tissue which had organized into fibrous tissue, together with the curvature of the spine, as the X-ray picture shows. The patient swallowed the upper suction plate with four front teeth attached while sleeping. The plate lodged in his throat, and a physician who was called in wished to push it down into the stomach, to which the patient objected. He then went to a hospital where, after the passage of several instruments, he was assured that he had never swallowed the plate or that it was no longer in the esophagus. For a few weeks after the patient felt slight pain in the lower part of the neck at the point he always felt that the object had lodged. After that time there was no sensation, but swallowing has always been difficult. The Kahler esophagoscope was used, and many attempts were made before this foreign body was successfully removed. By loosening the plate from the fibrous bed by setting up slight inflammatory action and then waiting a few days, the plate was removed without much ulceration of the structure.

